# Gestation Food Restriction and Refeeding Compensate Maternal Energy Status and Alleviate Metabolic Consequences in Juvenile Offspring in a Rabbit Model

**DOI:** 10.3390/nu13020310

**Published:** 2021-01-22

**Authors:** Rosa M. García-García, María Arias-Álvarez, Pilar Millán, María Rodríguez, Ana Sánchez-Rodríguez, Pedro L. Lorenzo, Pilar G. Rebollar

**Affiliations:** 1 Department of Physiology, Veterinary Faculty, Complutense University of Madrid, 28040 Madrid, Spain; pmillanp@vet.ucm.es (P.M.); anasanro.vet@gmail.com (A.S.-R.); plorenzo@vet.ucm.es (P.L.L.); 2 Department of Animal Production, Veterinary Faculty, Complutense University of Madrid, 28040 Madrid, Spain; m.arias@vet.ucm.es; 3 Department of Agrarian Production, ETSIAAB, Polytechnic University of Madrid, 28040 Madrid, Spain; maria_rodriguez_francisco@hotmail.com (M.R.); pilar.grebollar@upm.es (P.G.R.)

**Keywords:** food restriction, mother, fetus, offspring, metabolism, liver, rabbit model

## Abstract

Nutritional status during gestation can influence mother and offspring metabolism. Undernutrition in pregnancy affects women in both western and developing countries, and it is associated with a high prevalence of chronic diseases in later life. The present work was conducted in the rabbit model, as a longitudinal study, to examine the effect of food restriction during early and mid-gestation, and re-feeding ad libitum until the end of pregnancy on metabolic status and body reserves of mother and, its association with development and metabolism of fetuses and female offspring to the juvenile stage. Little changes in live body weight (LBW), compensatory feed intake, similar body reserves, and metabolism were observed in dams. Placenta biometry and efficiency were slightly affected, but fetal BW and phenotype were not modified. However, hyperinsulinemia, insulin resistance, and hypertriglyceridemia were demonstrated in pre-term fetuses. In the juvenile period, these changes were not evidenced, and a similar pattern of growth and serum metabolic parameters in offspring of food-restricted mothers were found, except in serum aminotransferases levels, which increased. These were associated with higher liver fibrosis. Maternal food restriction in the early and mid-pregnancy followed by re-feeding in our rabbit model established a compensatory energy status in dams and alleviated potential long-term consequences in growth and metabolism in the offspring, even if fetal metabolism was altered.

## 1. Introduction

According to the Developmental Origins of Health and Disease hypothesis, maternal food restriction during the intrauterine developmental period can affect long-term metabolic health of offspring due to alterations of organ structures and epigenetic programming [1,2] Maternal malnutrition influences on placenta development and efficiency, and subsequently, the availability of nutrients for the fetuses can be compromised [3,4]. In addition, fetal energy homeostasis [5] and metabolism [6,7] are reprogrammed and, as a result, the fetus re-distributes the limited fuel in order to ensure the development of vital organs (such as the brain and heart) by decreasing energy consumption in secondary organs (such as the liver, gut, etc.) [8]. This adaptation called “fetal programming” is beneficial for survival under undernourishment in the uterus, but detrimental when nutrition is abundant in post-natal life [9]. It highly depends on the timing of disturb takes place (i.e., embryogenesis, organogenesis, or organ growth) [10,11], and on the severity of the insult [12,13].

Most people are not concerned about the deep impact of nutrition during early pregnancy may have both on humans and livestock animals. In western countries, the strong desire to be thin lead the population to follow caloric restriction diets (about 30–50% of maintaining requirements) [14], included women of childbearing age [15], and it is observed that during pregnancy dietary patterns slightly change [16]. In addition, the number of undernourished women in developing countries is continuously rising, and recent economic and social crisis compromise the enhancement in these rates [17]. Thus, 9.7% of women in reproductive age (20–49 years) are underweight (body mass index-BMI 16–18.5) [18]. Due to low BMI, intrauterine growth retardation (IUGR) is provoked [9,19] and low birth weight children are born [20,21]. Compromised growth of a fetus in the uterus can induce a plethora of dysfunction of organs (kidney, liver, mammary gland, muscle, pancreas, and placenta) and/or organ systems (cardiovascular, adipose, nervous, endocrine, gastrointestinal, immune, and reproductive system). This situation is associated with a high prevalence of chronic diseases in later life [22], such as type 2 diabetes, obesity, cardiovascular disease, etc. [23,24], which has been considered as a global epidemic [25]. In addition, livestock animals show undernutrition commonly during gestation and some productive features can be also compromised as viability, growth, body composition, or metabolism (reviewed in [26]). Thus, additional attention to early-life environments is required.

Transgenerational studies make available an approach of setting the relative role of energy deficiencies during fetal life in the uterus, and the association with post-natal growth and development to later metabolic diseases [27], preventing adult diseases. Large scale studies in the fetus can be only achieved in animal models, and longitudinal experimental animal studies are also easier to perform in animals rather than humans. In this context, rodents, sheep, and pig have been investigated as animal models for developmental programming [11]. In the last decades, rabbits have also been used as an interesting animal model for these studies [6,28], due to their placentation type, which is similar to the human placenta, and the hemodynamic changes during pregnancy [29,30]. Regarding lipid metabolism, rabbits are more similar to humans than rodents [31]. A further advantage of studying rabbits is their larger size compared to rodents [32], but they are easier to manage in small animal facilities than sheep or pig.

Many studies have been conducted on overnutrition; however, less attention has been paid to the effects of undernutrition, and re-feeding on mothers and offspring metabolism. Nutritional interventions in undernourished pregnant mothers generally take place in the last trimester of gestation [32,33,34], in the form of supplements and nutritional support later in pregnancy [33]. In this context, the aim of the present work was to mimic the human situation of undernutrition during the two first third of gestation, followed by a dietary intervention in the last part of gestation. A better understanding of the complex metabolic interaction between the fetus and the mother may improve the metabolic health of the next generation, correcting disease trajectory. Then, we evaluated in the rabbit model, the influence of a maternal moderate food restriction during gestation followed by ad libitum re-feeding to term on: (1) the mother (feed intake, live body weight-LBW, and estimated body corporal composition, serum metabolic parameters, placenta morphometry, and efficiency); (2) pre-term fetus (morphometric parameters, sex ratio, and serum metabolic parameters), and (3) juvenile offspring (LBW, serum metabolic profile, and liver fibrosis). Considering this, our rabbit model showed that food restriction during gestation following by re-feeding at the last part of pregnancy and after birth, alleviated metabolic alterations observed in fetuses at pre-term and allow the development of metabolic healthy offspring until the juvenile stage. The translational value of our model was the possibility to study the metabolism and development of progeny from fetus to juvenile stage, associated with the mother metabolism, body reserves, and reproductive parameters.

## 2. Materials and Methods

### 2.1. Animals

New Zealand White × California rabbits were housed individually in flat-deck cages with constant light/darkness (16:8), 20 to 25 °C of temperature and 60 to 75% of relative humidity by a forced ventilation system. Animals had free access to fresh water. All the experimental procedures with animals were approved by the Animal Ethics Committee of the Polytechnic University of Madrid (UPM, Spain) (PROEX 302/15), and were in compliances with the Spanish guidelines for care and use of animals in research (BOE, 2013), according to European Union Regulation 2010/63/UE.

### 2.2. Experimental Design

#### 2.2.1. Dams

**Figure 1:**
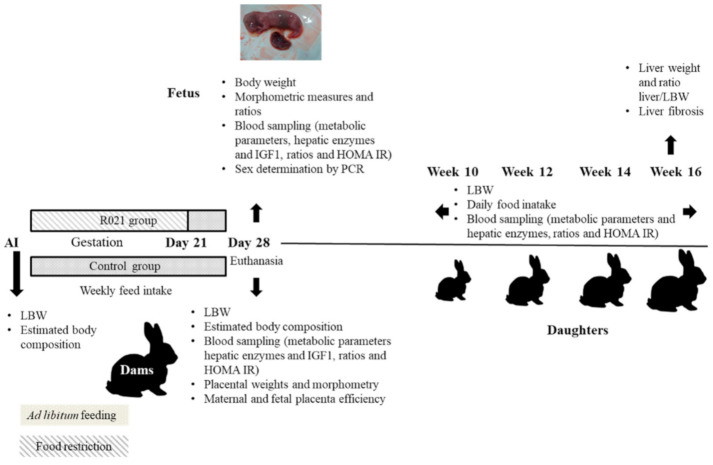
Experimental design. Mothers of R021 group were food restricted from Day 0 (artificial insemination, AI Day) to Day 21 (105 g/day) that means the first two-thirds of gestation, and ad libitum fed during the last third of gestation (gestation length is 31 days). Control group was fed ad libitum all the gestation period. Sampling time for dams was Day 0 and Day 28 (pre-term). For offspring, sampling time was on Day 28 of gestation (fetuses) and, from week 10 to 16 (juvenile phase), when assessments were taken each two weeks. LBW: live body weight; IGF1: insulin growth factor 1; HOMA-IR: homeostatic model assessment.

Rabbit females were fed ad libitum with a commercial diet (2400 kcal de ED/kg, 35% FND y 16% PB, NANTA, Madrid, Spain) during their first pregnancy. At that moment, their feed intake was recorded daily and established in a mean of 175g per animal and day. Then, animals were artificially inseminated (AI) again after weaning (30 days post-partum) with fresh diluted semen (MA 24, Ovejero, León, Spain). Ovulation was induced with gonadorelin at the time of AI (20 μg/doe, i.m.; Inducel-GnRH, Ovejero, León, Spain). At this moment, a total of 121 does were randomly allocated into two groups: Control group (*n* = 61) fed ad libitum during all pregnancy, and R021 group (*n* = 60) restricted to 60% of their previous daily food intake (105 g/day), from Day 0 to Day 21 of pregnancy, being the duration of the gestation of 31 days. Animals of R021 group were fed ad libitum the last week of pregnancy and until the end of the study (Figure 1). Does feed intake was weekly determined in a sample of 33 females of control group and 47 mothers of R021 group.

At Day 28 of pregnancy, 7 does from each group were chosen randomly from those diagnosed as pregnant by abdominal palpation. LBW and estimated body composition at Day 0 (AI day) and at pre-term (Day 28) were determined. Body composition was estimated by means of bioelectrical impedance analysis (BIA) using a four-terminal body composition analyzer (Model Quantum II, RJL Systems, Detroit, MI, USA), as described by Sakr et al. [35]. Blood samples were collected from the ear vein into heparinized tubes and centrifuged for 15 min and 700× *g* at 4 °C, and plasma stored to −80 °C. Glucose, insulin, triglycerides (TG), cholesterol, serum alanine transaminase (ALT) and aspartate transaminase (AST), and insulin growth factor 1 (IGF1) concentrations were determined, and insulin/glucose, TG/cholesterol, and AST/ALT ratios were calculated. According to Menchetti et al. [6], the presence of insulin resistance was evaluated through homeostasis model assessment for insulin resistance (HOMA-IR). It was calculated using the formula [insulin (mU/L) × (glucose (mg/dL)/18)]/22.5. Immediately after blood sampling, the same does were euthanized (30 mg/kg; Dolethal, Madrid, Spain) to remove the gravid uterus by a medial ventral laparotomy. Fetuses were dissected from their extraembryonic membranes and classified in: viable (with a correct morphology and weight for the gestational age), non-viable (mummified: dead in uterus with signs of shriveling and drying), and reabsorbed (fetoplacental residues adhering to the endometrium without the appearance of a fetus). Total fetuses and viability rate per doe [(number of viable fetuses/number total of fetuses) × 100] were recorded. Using scales, total placenta weight and weights of decidua (maternal placenta) and labyrinth zone (fetal placenta), previously separated, were recorded in viable fetuses from the two groups. In addition, length, breadth, and thickness of both placentas were measured by means of calipers. Total placenta efficiency (fetus weight/total placenta weight), maternal and fetal placenta efficiency (fetus weight/maternal placenta weight and fetus weight/fetal placenta weight, respectively) were calculated.

#### 2.2.2. Fetuses

In all viable recovered fetuses (*n* = 157), body weight (BW), crown-rump length, biparietal and thoracic diameters were determined using scales and calipers. Head, trunk, liver, gut, and brain weights were recorded by means of a scale. Relative brain and liver weights to fetal BW, and brain to liver weight ratios were calculated to assess fetal growth pattern.

After that, the next determinations and analysis were made in a subset of 60 fetuses from control (*n* = 30) and R021 group mothers (*n* = 30). Offspring blood was obtained after decapitation by collecting samples in tubes without anticoagulant and stored at 4 °C overnight. After that, serum was recovered and stored at −20 °C until further analysis of metabolic parameters (glucose, insulin, TG, cholesterol, ALT, and AST) and IGF1 concentrations. In addition, insulin/glucose, TG/cholesterol, AST/ALT ratios, and HOMA-IR index were calculated, as previously stated. Fetuses were sexed by PCR in fetal liver samples to assess sex ratio.

#### 2.2.3. Offspring

Offspring of all females after weaning (4th weeks of age) were fed ad libitum with the same diet as their mothers until the end of the study. From 10 weeks until the age of 16 weeks (juvenile stage), the daughters from randomly chosen females belonging to control (*n* = 13) and R021 group (*n* = 17) were housed in individual cages, and LBW and feed intake were determined biweekly. Furthermore, blood samples were obtained from the ear vein in tubes with anticoagulant, centrifuged for 15 min and 700 g at 4 °C, and plasma stored to −80 °C until analysis. Glucose, insulin, TG, cholesterol, ALT, and AST were determined, and ratio insulin/glucose, TG/cholesterol and AST/ALT, and HOMA IR index were calculated. Finally, at 16 weeks of age all daughters were euthanized (30 mg/kg; Dolethal, Madrid, Spain) and their liver was recovered and weighted; the ratio liver weight/LBW was determined. Study of the collagen content in the liver was carried out by Sirius red staining.

### 2.3. Blood Analysis

Parameters relating to the carbohydrates metabolism (glucose), lipid metabolism (to-tal cholesterol and TG), and hepatic profile (ALT and AST) were measured according to the instructions of commercial kits (Biolabo, Maizy, France), on a clinical biochemical analyzer (Tecom Science Co., Nanchang, China). Concentrations of hormones IGF1 and insulin were determined using specific commercial EIA kits (IGF1: Demeditec Diagnostics GmbH, Kiel, Germany and insulin: Mercodia AB, Uppsala, Sweden). The assay sensitivity was 0.091 ng/mL and 0.15 mU/L, respectively. The intra-assay variation coefficient was 5.81% and 3.90%, and the inter-assay variance was 8.57% and 5.30% for IGF1 and insulin, respectively.

### 2.4. Sex Determination by PCR

Sexing of fetuses was carried out by PCR in fetal livers samples. A piece of liver from the right lobe was recovered and snap-frozen in liquid nitrogen. Afterwards, it was stored at −80 °C until analysis.

For PCR, DNA was extracted with DNAzol^®^ Reagent (Invitrogen-Thermo Fisher Scientific, Waltham, MA, USA) following the instructions of the manufacturer. Then, DNA was quantified using the NanoDrop ONE spectrophotometer (Thermo Fisher Scientific, Waltham, MA, USA). Primers OcSRY (F: AGCGGCCAGGAACGGGTCAAG and R: CCTTCCGGCGAGGTCTGTACTTG) and OcGAPDH (F: TGAACGGATTTGGCCGCATTG and R: ATGCCGAAGTGGTCGTG-GATG) were used for amplification according to Vašíček et al. [36], with the following PCR conditions: 94 °C 2 min, 94 °C 20 s, 64 °C 30 s, 72 °C 30 s, 72 °C 10 min in 35 cycles [37]. The reaction mixture contained a total of 200 ng DNA, 2 mM MgCl_2_, 0.4 µM of each primer, 200 µM dNTP, and 0.625 U AmpliTaq Gold DNA Polymerase (Applied Biosystems, Waltham, MA, USA). PCR products (4 µL) were checked on 2% agarose gel stained with GelRed™ (Biotium, San Francisco, CA, USA) and low DNA Mass Ladder (Invitrogen Thermo Fisher Scientific, Waltham, MA, USA) was used for verifying the expected size of the product. They were visualized by BioDoc-It Imaging system (UVP, Upland, CA, USA).

### 2.5. Liver Fibrosis by Sirius Red Analysis

Small pieces of liver about 0.5–1 cm long from the right lobe of 16th weeks daughters were fixed in 4% neutral buffered paraformaldehyde (in PBS, pH 7.0) for 24 h at room temperature. Following dehydration in a series of ethanol and xylene steps, samples were embedded in paraffin and were cut in 7 μm-thick sections. Slides were stained with Sirius red. For Sirius Red staining, the sections were dewaxed, hydrated and stained with 1% Picro-Sirius Red (Sigma Aldrich, Saint Louis, MO, USA) for 1 h. Then, the specimens were washed, dehydrated in 100% ethanol, cleared, sealed, and observed under a light microscope (Axioplan-2, Zeiss, Germany). Collagen fibers appeared red on a pale-yellow background. The degree of red staining is well correlated with collagen content and with hepatic fibrosis [38].

Five fields were randomly taken for three sections of each liver (cut each 200 µm) with MetaMorph software (Molecular Devices, San Jose, CA, USA) at 10× magnification. A binary image was produced and the total area of collagen and the hepatic proportional fibrotic area (expressed as a percentage of the fibrotic area to total whole analyzed area) were measured according to Mori et al. [39].

### 2.6. Statistical Analysis

Statistical analyses were performed with SAS software (SAS Inst. Inc., Cary, NC, USA). The experimental unit was the mother in the study of dams. Weekly feed intake of pregnant does, LBW, and estimated body composition of does at Day 0 and Day 28 were assessed by repeated measures analysis (MIXED procedure), with nutrition levels (ad libitum and food restriction), examination days (weekly in feed intake or Day 0 and Day 28 of pregnancy in LBW), and their interaction taken as main effects. Metabolic parameters of does and morphometric parameters of placenta and fetuses (with the number of total fetuses present in uterus included as a covariate) on Day 28 of pregnancy were analyzed as a completely randomized design, with food restriction as the main source of variation, using the GLM procedure.

In the study of fetuses, the fetus was considered as the experimental unit. The differences in serum metabolites, insulin, and IGF1 concentrations were analyzed considering the nutritional restriction and the sex as the main effects, as well as the corresponding interaction. Fetal sex remained in the model only when the effect was significant.

In the juvenile stage, each daughter from randomly chosen mothers was considered the experimental unit. LBW, feed intake, and metabolic parameters were analyzed by repeated measures (MIXED procedure), with dietary restriction, measurement time (bi-weekly), and their interaction as main effects. The fibrosis area and the percentage of collagen fibers in the liver were analyzed with food restriction as the main source of variation, using the GLM procedure.

All means were compared using a protected *t*-test. Differences were considered significant at *p* < 0.05 and a trend when *p* < 0.10. Results are presented as least squared mean (ls means ± S.E.M.).

## 3. Results

### 3.1. Maternal Response to Food Restriction during Gestation

#### 3.1.1. Feed Intake, LBW, and Estimated Body Composition

**Figure 2:**
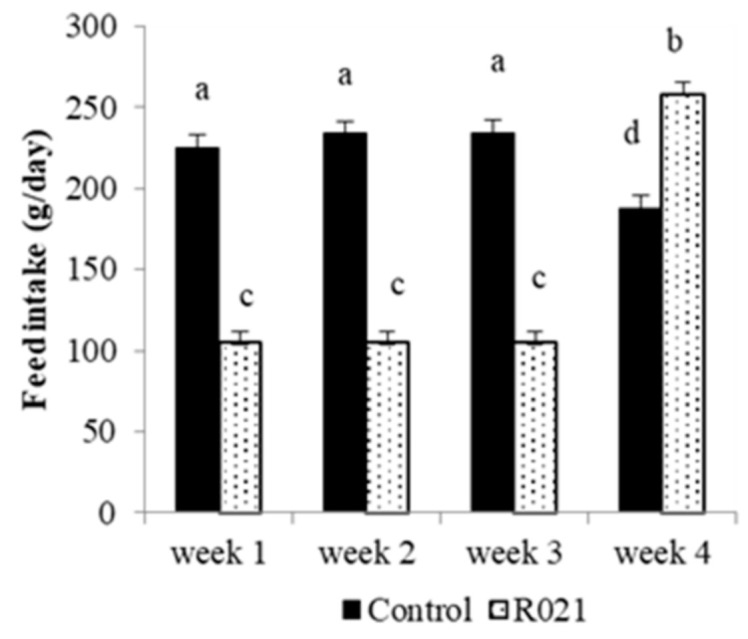
Feed intake (g/day) of pregnant does fed ad libitum (Control group; *n* = 33) or food restricted (105 g/day) during the two-third of gestation and re-fed ad libitum until term (R021 group; *n* = 47). Length of gestation is around 31 days. Different letters indicate significant differences between groups and weeks (*p* < 0.05).

Due to the experimental design, feed intake was different between experimental groups during the first three weeks (*p* < 0.05; Figure 2). However, in the last week of pregnancy, animals that had been restricted significantly increased (*p* < 0.05) their voluntary feed intake, even above the consumption of the control group the previous weeks. In addition, feed intake markedly fell during the final days of pregnancy in females of the control group, just as expected in this phase.

As shown in Table 1, similar LBW and estimated body composition were recorded between food restricted and control females, although significant differences (*p* < 0.05) were observed from Day 0 to Day 28 of pregnancy in both experimental groups.

#### 3.1.2. Biochemical and Hormonal Serum Analysis of Does

Food restriction did not affect plasma metabolic and hormonal parameters at Day 28 of gestation (Table 2). Furthermore, calculated ratios were similar for both groups.

#### 3.1.3. Morphometric Study of Placental Structures

Placental development was slightly affected in the fed restricted group (Table 3). Total and fetal placenta efficiency tended to be lower for undernourished females (*p* = 0.0751 and *p* = 0.0684, respectively) and, only decidua and labyrinth zones length was significantly lower for R021 group (*p* < 0.05). No other differences were observed between groups within the remaining macroscopic parameters measured.

### 3.2. Fetal Response to Maternal Food Restriction during Gestation

#### 3.2.1. Morphometric Study of Fetuses and Sex Ratio

All the values for morphometric measurements and ratios of fetuses were similar in both groups (Table 4).

**Figure 3:**
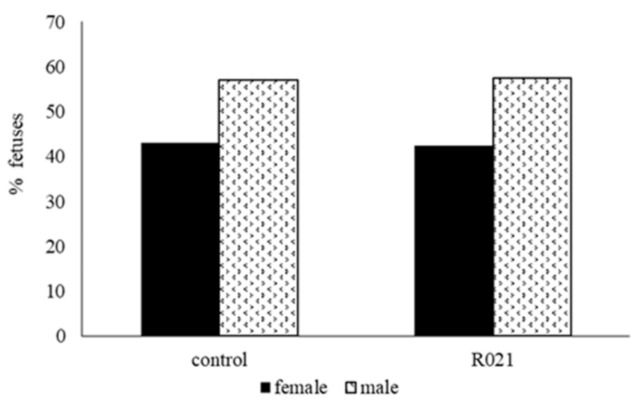
Proportions of females and males at 28 days of gestation from ad libitum fed mothers (Control group; *n* = 30) and fed restricted females (105 g/day) during the first two-thirds of gestation and re-fed ad libitum until term (R021 group; *n* = 30). Day 0, day of artificial insemination (AI); length gestation, 31 days.

Sex ratio was unbiased by the maternal food restriction during gestation (Figure 3).

#### 3.2.2. Metabolic and Hormonal Parameters

**Figure 4:**
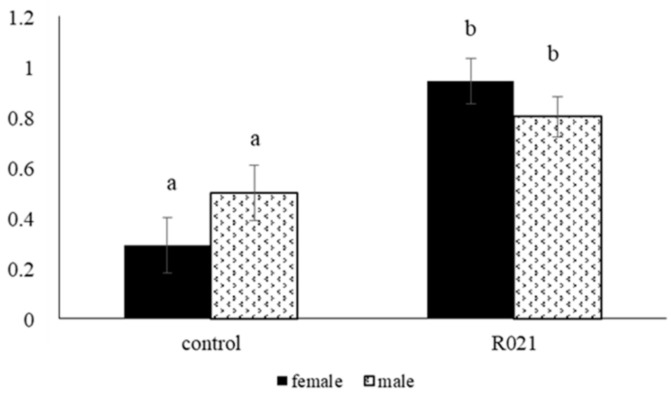
HOMAR IR index in female and male fetuses on Day 28 of gestation of ad libitum (Control group) and fed restricted females (105 g/day) during the first two-thirds of gestation and re-fed ad libitum until term (R021 group). Day 0, day of artificial insemination (AI); length gestation, 31 days. Different letters indicate significant differences between groups (*p* < 0.05). HOMA index: [insulin (mU/L) × (glucose (mg/dL)/18)]/22.5.

Fetuses of fed restricted mothers showed significantly higher insulin levels (*p* = 0.0063), and HOMA IR index (*p* < 0.0001), and tended to have a higher ratio insulin/glucose (*p* = 0.06) than those of control ones (Table 5). In addition, females of the restricted group compared to females of the control group showed higher insulin concentrations (8.02 ± 1.67 vs. 3.00 ± 1.30 mU/L; *p* = 0.0325) and tended to have a higher ratio insulin/glucose (0.18 ± 0.03 vs. 0.09 ± 0.03; *p* = 0.0979). The interaction between sex by treatment denoted a trend (*p* = 0.0982) for HOMA IR index, being higher for both sexes from restricted group than those from control (Figure 4).

Serum TG concentrations significantly increased (*p* = 0.0347), and the ratio TG/cholesterol tended to be higher (*p* = 0.0940) for the fetuses of undernourished dams (Table 5). In addition, males from restricted group presented higher TG levels (116.75 ± 6.93 vs. 90.72 ± 6.06 mg/dL, *p* < 0.0069) and ratio TG/cholesterol (1.04 ± 0.06 vs. 0.83 ± 0.06, *p* = 0.0248) than control males. No differences in glucose concentration, total cholesterol concentration, and enzyme AST activity were observed between groups. In the present work, the enzyme ALT was undetectable in fetal serum. IGF1 concentrations in serum of fetuses from underfed mothers were similar than those of fetuses from control dams. Within the groups, we found no significant differences between male and female fetuses.

### 3.3. Influence of the Maternal Food Restriction during Gestation in the Juvenile Phase (from 10 to 16 Weeks of Age)

#### 3.3.1. LBW, Feed Intake, Serum Metabolic and Hormonal Parameters, and Hepatic Enzymes

**Figure 5:**
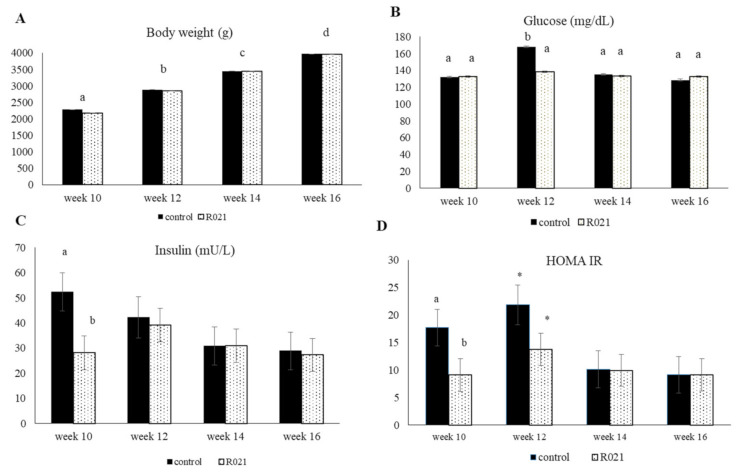

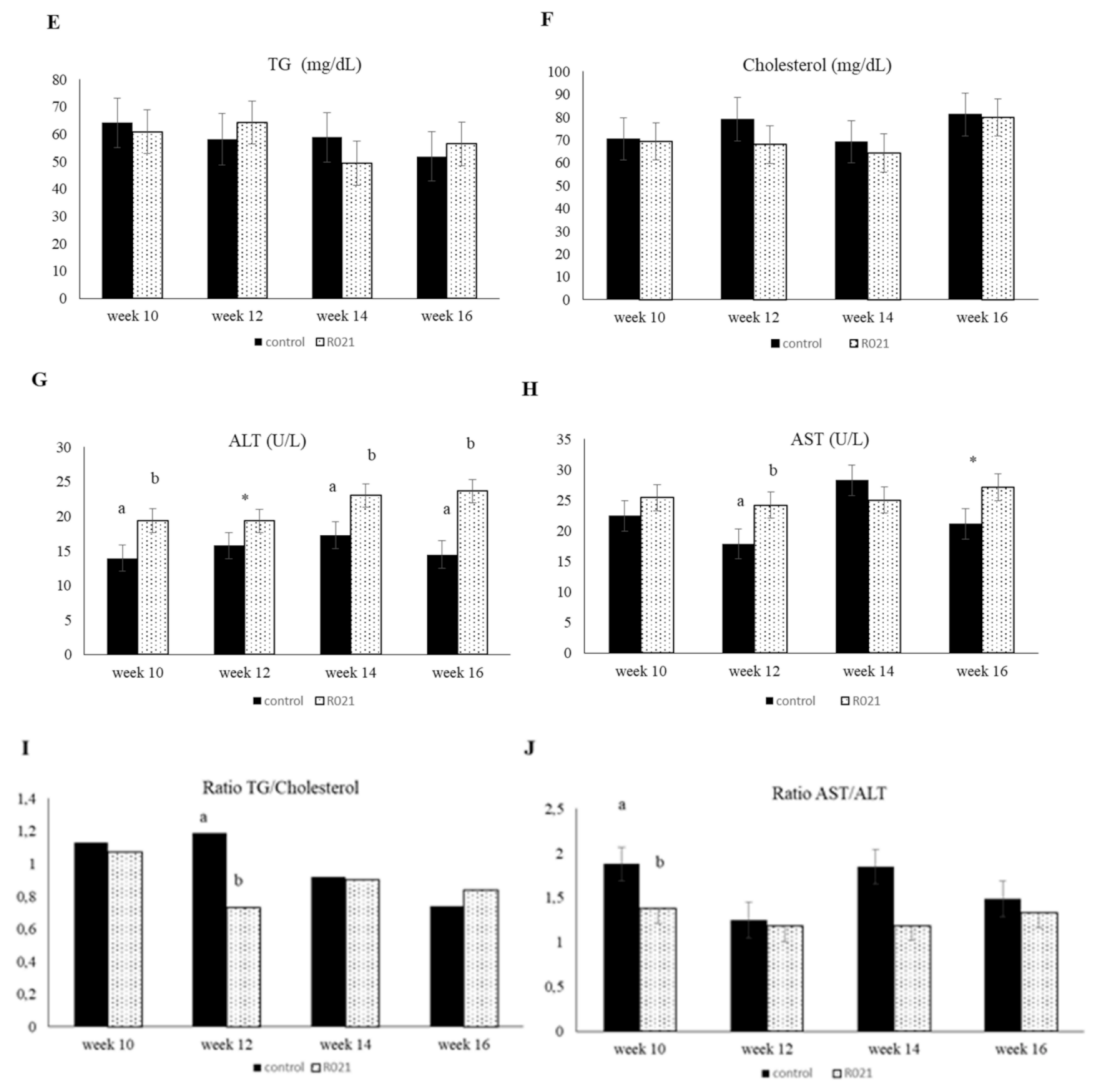
Live body weight (LBW) and metabolic parameters during the juvenile phase (10 to 16 weeks) of female progeny from ad libitum fed mothers (Control group) and fed restricted females (105 g/day) during the first two-thirds of gestation and re-fed ad libitum until term (R021 group). (**A**) Live body weight (LBW); carbohydrate metabolites: (**B**) glucose and (**C**) insulin; (**D**) HOMA IR index; lipid metabolism: (**E**) triglycerides, (**F**) cholesterol and (**I**) ratio TG/cholesterol; hepatic enzymes: (**G**) ALT, (**H**) AST and (**J**) ratio AST/ALT. Different letters mean significant differences (*p* < 0.05) between groups within weeks, except in LBW that denote differences between weeks. HOMA IR index: [insulin (mU/L) × (glucose (mg/dL)/18)]/22.5; ALT: serum alanine transaminase; AST: serum aspartate transaminase. * denotes *p* = 0.01.

From 10 to 16 weeks, mean daily feed intake of daughters was similar for control and R021 group (214.86 ± 9.65g vs. 226.70 ± 8.44 g). LBW of daughters were unaffected by mother undernourishment during pregnancy, but as expected, LBW progressively increased during the period observed (*p* < 0.0001) (Figure 5A). For plasma metabolic concentrations, offspring showed similar levels except for hepatic enzyme ALT, which were significantly higher for daughters from restricted mothers (*p* = 0.0011; Figure 5G). The ratio AST/ALT was significantly lower in R021 group in week 10 (*p* = 0.0539) and week 14 (*p* = 0.014) (Figure 5J) and AST levels increased in week 12 (*p* = 0.0566) and tend to be higher in week 16 for R021 group (*p* = 0.0775) (Figure 5H). HOMA IR index was affected by restriction (*p* = 0.0561) and by time (*p* = 0.0403), mainly because in week 12 control females showed significantly higher HOMA IR index than animals in later weeks, where HOMA-IR index was stabilized (Figure 5D). The rest of the parameters was maintained during all the juvenile period, with only significant changes in glucose concentrations at week 12 (*p* = 0.0008) (Figure 5A).

#### 3.3.2. Liver Weight and Fibrosis at 16 Weeks

Female offspring from restricted dams at 16 weeks of age showed similar liver weight (168.82 ± 9.01 vs. 153.97 ± 8.12g) and liver-to-body weight ratio (3.98 ± 0.21 vs. 3.67 ± 0.19) comparable to those from the control group mothers.

Liver of the juvenile females displayed a completed structure and a normal appearance. There was fibrosis formation in the portal area in both groups, but moderate fibrous septa forming in the lobule were found for R021 group (Supplementary Figure S1). Fibrosis area (106505.27 ± 5694.43 vs. 80652.25 ± 6384.73 pixels/µm2; *p* = 0.0027) and percentage of collagen (6.82 ± 0.36 vs. 5.17 ± 0.40%; *p* = 0.0029) was significantly higher for R021 group than for control group.

## 4. Discussion

The malnutrition during gestation in some parts of child-bearing age population should be a public concern and interventions to improve health of mothers and progeny are required. The present work demonstrates that in the rabbit model, maternal undernutrition throughout the first two-thirds of gestation and re-feeding with ad libitum diet to term alleviates metabolic changes in dams and juvenile progeny, although metabolism of fetuses is compromised. Moderate maternal feed restriction provoked mild changes in LBW and metabolism of females does, as well as in its placental biometry and efficiency. Fetal BW was not affected and no IUGR could be observed with this food restriction level. However, some changes in the metabolism of insulin (hyperinsulinemia and insulin resistance) and lipids levels in serum (hypertriglyceridemia) were demonstrated in pre-term fetuses. These reprogramming changes were not exhibited in the juvenile phase of the female offspring fed ad libitum in the post-natal period. Likewise, growth, LBW, and serum metabolic parameters were similar in offspring from control and restricted mothers, except for hepatic transaminases that denoted certain liver damage, confirmed by higher liver fibrosis in offspring of restricted dams.

Feed intake of underfed mothers increased during the last week of gestation, when feeding was re-established, according to Daoud et al. [40] and Lopez Tello et al. [4]. Probably, this compensatory feed intake during the last third of gestation was enough to promote recuperation of the maternal LBW and energetic body reserves, as shown in estimated body composition results. Both groups presented lower fat and protein content as usual at the end of pregnancy in animals [41] and humans [41,42]. Consequently, unaffected LBW in dams was related to no significant changes in glycemic metabolism (glucose, insulin, and ratio insulin/glucose) and lipid metabolism (TG, cholesterol, and ratio TG/cholesterol) at the end of pregnancy. Rabbit does did not show insulin resistance assessed by HOMA IR index, in contrast to other studies in underfed rat mothers during early gestation [43]. These authors observed that undernutrition during the first half of pregnancy exacerbated the insulin-resistant state at the end of gestation, as physiologically occurs in women. In the current work, glucose levels were lower at term in R021 group, maybe due to the higher expense of energy required for the growing fetuses [44,45]. Similar results were found when the nutritional restriction was performed at mid- and late pregnancy in rabbits [46] and sheep [47]. Taken together these findings, the nutritional intervention in the last phase of pregnancy may favor a physiological condition in the undernourished pregnant mother, alleviating adverse metabolic conditions. This is positive since energetic substrates in the mother are regulating fetal substrate availability [48].

The slight decrease in total placental efficiency and placenta size in fed restricted mothers did not result in low fetal weight and impairment of fetal morphometric measurements. Severe alterations in placental and fetal development have been observed in previous works of our group when maternal food restriction (50% of maintenance requirements) is maintained during all gestation time in the rabbit model. Histological changes in placenta of undernourished females were found. Fewer vessels, with hyalinized and sclerotic walls, and lesser supporting stroma as well as necrosis were observed [4]. In addition, Fortun et al. [49] also appreciated lower weight rates when does were restricted to 75% of their maintenance requirements during the entire pregnancy, but they observed a similar rate of embryo viability. Fetal growth is lower only when food supply is severely diminished [50]. In the current work, we exposed animals to nutritional restriction during the first two-thirds of pregnancy at a similar level than found in regimes in both humans and animals [51]. In these periods, embryo development (0–7 days) and organogenesis (7 to 18 or 19 days) are carried out in the rabbit [52]. It was shown that undernutrition during the first half of gestation less affected the birth weight [53], although metabolic function could be influenced in the progeny [54]. When maternal nutrient restriction is set in late gestation, reduced offspring birth weight was shown in rat [55], cow [56,57], and sheep [58]. In addition, those differences in fetal body weight during late gestation are associated in the sheep model with altered fetal blood pressure trajectories [59]. The explanation for that may be the exponential fetal growth that happens in the last period of gestation, when the fetus may be more susceptible to nutritional restriction than in the early and mid-gestation phase [60]. Probably, in the present work re-feeding the mothers in the last week allowed fetuses to caught development of their control counterparts. It did not provoke any severe impairment in body growth and organ development, as previously described [11,50], even if preimplantation and organogenesis periods were compromised in these offspring. In fact, fetal reprogramming was shown in some metabolic substrates in the serum of undernourished fetuses and insulin resistance appeared for fetuses of R021 group.

Availability of lipids and insulin mainly depends on de novo synthesis by the fetus more than maternal transfer by the placenta [61]. Fetuses of undernourished dams exhibited an increase in insulin serum concentration and higher ratio insulin/glucose, as well as higher HOMA IR index. Increased insulin levels in serum were higher for R021 female fetuses than for control group females, as occurs in sheep [62], indicating specific sensitivity for the female sex than for the male sex. All these findings denoted insulin resistance since more insulin per glucose unit is required to maintain blood glucose levels in the physiological range. This higher insulin level may be a response to maintain glucose level in physiological concentration in blood and body tissues [63], and to meet fetal nutrient requirements in anticipation of a further reduction in fetal substrate supply [64]. Hence, the increased insulin level decreased gluconeogenesis [64,65] and induced the increase of serum TG by de novo synthesis of lipids in the liver [44,66]. TG will be stored and can serve for gluconeogenesis [67]. In the present study, higher circulating levels of serum TG and a tendency for higher ratio TG/cholesterol were seen in the undernourished fetuses. Total cholesterol was unaffected. Other studies have also found an increase in fetal serum TG levels (beef: [68]; sheep: [69]; pig: [61]), and even this response is shown when the fetus is challenged with other insults, such as environmental pollution [70]. Hypertriglyceridemia supported the impaired glucose tolerance found in fetuses of undernourished dams since the level of serum TG is accepted as a good indicator of insulin resistance [71]. These metabolic adaptations set the scene for fuel deficiency [72]. In addition, we found a sex-related effect in serum TG concentrations, which were higher in males from undernourished dams. Current findings denoted more sensitivity for lipid metabolism of TG in male fetuses according to other works [58,66] and in contrast with those of Zhou et al. [5]. These authors reported lower serum TG levels in the male offspring from restricted goats during mid- and late pregnancy. Such controversy could be explained because lipid metabolism appears early in the development of undernourished male fetuses [73]. Sex specificity of metabolic outcomes is a common finding in the literature [5,25]. However, in the current work undernutrition did not result in sex-specific changes in body and organ weights, morphometric parameters, body composition, and only some metabolic parameters were affected in the fetal period.

Despite the hyperinsulinemia and hypertriglyceridemia exhibited by fetuses of R021 group, that may increase the susceptibility to fatty liver, type 2 diabetes, and obesity during adulthood [22], in the present work the studied offspring did not exhibit any remarkable alteration in growth and food consumption during juvenile phase until puberty. These results agree with those of Symmeon et al. [74], who reported similar body weight and feed intake during fattening in rabbit offspring of undernourished dams and, with Hyatt et al. [10] in juvenile sheep undernourished during early to mid-gestation. However, Borwick et al. [58] showed lower weight at weaning and lower growth in the progeny of restricted sheep mothers during late gestation. Female offspring from underfed mothers did not develop glucose intolerance from 10- 16 weeks of age, and even HOMA IR index was lower than their control counterparts, showing low insulin resistance. From birth onwards, all animals were fed ad libitum, and probably this intervention compensated reprogramming changes in the uterus. In 3 month-kids of restricted goats during mid- and late pregnancy, ad libitum food intake after birth also alleviated liver metabolic changes observed in the fetuses [5].

In juvenile female offspring we found increased fibrosis without damage of hepatic lobule, according to elevated ALT and AST levels. These increased concentrations of amino transaminases, which are clinical indicators of liver function [75,76], also in rabbits [77,78], were probably related to histopathological changes in the liver [79]. Generally, ALT level rise is higher than AST level when both are elevated, because of the longer half-life of ALT and the more significant fraction of AST that is bound to the mitochondria [80]. Increased ALT levels reveal hepatocellular damage and are strongly correlated with liver fat content [81], being commonly used as a marker of NAFLD (non-alcoholic fatty liver disease). Low protein diet in rats showed a similar pattern with increased hepatic enzyme levels in offspring adult male rats [82]. Then, our results suggest that altered metabolic parameters found in fetuses could be reverted to physiological levels when progeny is fed ad libitum after birth, although some hepatic changes persist. Therefore, life-long signals and tissue-specific epigenetic alterations are probably present. According to other authors [48,60,79] possible restoration of energy demands at the end of gestation in dams and normal feeding of the newborn can ease reprogramming events and may improve offspring outcomes.

## 5. Conclusions

The present work states that moderate food restriction during early and mid-gestation followed by ad libitum re-feeding in the late gestation, lead to a compensatory feed intake in dams that attenuates changes in their LBW and body reserves in the rabbit model. Some adverse alterations in placenta do not modify the fetal phenotypic response to intrauterine supply restriction, but an insulin-resistant state and hypertriglyceridemia in fetuses are evidenced. Female rabbit offspring does not exhibit alterations in growth and metabolic status until the juvenile stage, but some liver damage is found. Then, even if mothers have low food consumption during gestation, an appropriate feeding regime during late gestation could improve metabolic status in mothers, and consequently, offspring metabolism and development can be favored.

## Figures and Tables

**Table 1:** Effect of time (Day 0 and Day 28 of pregnancy) and maternal food restriction (MFR) on live body weight (LBW) and estimated body composition of ad libitum (control group) and fed restricted females (105 g/day) during the first two-third of gestation and re-fed ad libitum until term (R021 group). Day 0, day of artificial insemination (AI); length of gestation, 31 days.

	Control (*n* = 7)		R021 (*n* = 7)		*P*_time_ > f	*P*_MFR_ > f	*P*_timexMFR_ > f
	Day 0	Day 28	Day 0	Day 28			
LBW (g)	3990 ± 122.0	4692 ± 122.0	4049 ± 86.6	4608 ± 86.6	0.0001	0.9288	0.1989
Water (%)	58.8 ± 1.75	88.0 ± 1.75	58.8 ± 1.24	86.8 ± 1.24	0.0001	0.6609	0.7218
Ash (%)	3.30 ± 0.05	3.06 ± 0.05	3.30 ± 0.03	3.08 ± 0.03	0.0003	0.8481	0.8036
Lipids (%)	15.4 ± 1.78	11.9 ± 1.78	15.3 ± 1.26	12.1 ± 1.26	0.0835	0.9780	0.9454
Proteins (%)	19.36 ± 0.13	17.6 ± 0.13	19.38 ± 0.09	17.7 ± 0.09	0.0001	0.7102	0.7530
Energy (MJ/kg)	147.0 ± 80.6	955.7 ± 80.6	1144.0 ± 57.0	966.04 ± 57.0	0.0439	0.9518	0.9350

**Table 2:** Metabolic, hormonal parameters, and hepatic enzymes levels on Day 28 of gestation of ad libitum (Control group) and fed restricted females (105 g/day) during the first two-thirds of gestation and re-fed ad libitum until term (R021 group). Day 0, day of artificial insemination (AI); length gestation, 31 days.

	Control (*n* = 7)	R021 (*n* = 7)	*p* > f
Glucose (mg/dL)	119.4 ± 8.29	102.3 ± 8.29	0.1715
Insulin (mU/L)	8.37 ± 2.10	12.34 ± 2.10	0.2078
Ratio glucose/insulin	18.1 ± 6.05	16.2 ± 2.10	0.8308
HOMA IR index ^1^	2.46 ± 0.52	2.90 ± 0.52	0.5602
TG (mg/dL)	78.6 ± 12.9	88.2 ± 12.9	0.6096
Total cholesterol (mg/dL)	31.6 ± 4.62	23.9 ± 4.62	0.2597
Ratio TG/cholesterol	2.97 ± 0.72	4.06 ± 0.72	0.3074
AST(U/L)	58.4 ± 20.3	103 ± 20.3	0.1461
ALT (U/L)	9.14 ± 6.11	23.6 ± 6.11	0.1194
Ratio AST/ALT	6.77 ± 1.32	5.64 ± 1.32	0.5553
IGF1 (ng/mL)	267.9 ± 26.1	296.4 ± 26.1	0.4621

^1^: HOMA index: [insulin (mU/L) × (glucose (mg/dL)/18)]/22.5; TG: Triglycerides; ALT: serum alanine transaminase; AST: serum aspartate transaminase; IGF1: insulin growth factor 1.

**Table 3:** Placenta morphometric characteristics and efficiency on Day 28 of gestation of *ad libitum* (Control group) and fed restricted females (105 g/day) during the first two-thirds of gestation and re-fed *ad libitum* until term (R021 group). Day 0, day of artificial insemination (AI); length gestation, 31 days.

	Control (*n* = 7)	R021 (*n* = 7)	*p* > f
Total placenta weight (g)	4.99 ± 0.14	5.08 ± 0.12	0.6785
Total placenta efficiency (%)	7.84 ± 0.19	7.35 ± 0.17	0.0751
Decidual zone			
Weight (g)	1.44 ± 0.05	1.42 ± 0.05	0.8493
Length (mm)	40.9 ± 0.65	35.8 ± 0.56	0.0001
Thickness (mm)	3.25 ± 0.12	3.28 ± 0.10	0.8545
Maternal placenta efficiency	28.2±1.14	27.3±1.00	0.5907
Labyrinth zone			
Weight (g)	3.53 ± 0.12	3.57 ± 0.11	0.8384
Length (mm)	38.8 ± 0.63	34.3 ± 0.55	0.0001
Thickness (mm)	4.91 ± 0.13	5.23 ± 0.14	0.1727
Fetal placenta efficiency	11.4 ± 0.30	10.6 ± 0.26	0.0684

**Table 4:** Morphometric parameters of viable fetuses on Day 28 of gestation of ad libitum (Control group) and fed restricted females (105 g/day) during the first two-third of gestation and re-fed ad libitum until term (R021 group). Day 0, day of artificial insemination (AI); length gestation, 31 days.

	Control (*n* = 7)	R021 (*n* = 7)	*p* > f
Morphometric measurements			
Biparietal diameter (mm)	19.4 ± 0.15	19.1 ± 0.13	0.2291
Crown-rump length (mm)	100.7 ± 0.76	99.3 ± 0.68	0.1998
Thoracic diameter (mm)	20.9 ± 0.30	20.6 ± 0.27	0.4534
Fetus weights			
Total (g)	39.4 ± 0.74	38.2 ± 0.67	0.2733
Head (g)	9.45 ± 0.15	9.23 ± 0.13	0.3245
Trunk (g)	28.5 ± 0.59	27.8 ± 0.52	0.4302
Liver (g)	2.52 ± 0.10	2.40 ± 0.08	0.3940
Gut (g)	1.89 ± 0.06	1.90 ± 0.05	0.8697
Brain (g)	0.91 ± 0.02	0.93 ± 0.01	0.4239
Weight ratios			
Brain ratio (%)	2.36 ± 0.06	2.48 ± 0.05	0.1385
Liver ratio (%)	6.40 ± 0.17	6.49 ± 0.14	0.7288
Brain: Liver ratio (%)	37.9 ± 1.98	39.9 ± 1.66	0.4546

**Table 5:** Metabolic and hormonal serum parameters and hepatic enzymes levels of fetuses on Day 28 of gestation of ad libitum (Control group) and fed restricted females (105 g/day) during the first two-thirds of gestation and re-fed ad libitum until term (R021 group). Day 0, day of artificial insemination (AI); length gestation, 31 days.

	Control (*n* = 30)	R021 (*n* = 30)	*p* > f
Glucose (mg/dL)	46.26 ± 3.16	49.70 ± 3.99	0.5028
Insulin (mU/L)	3.80 ± 0.77	7.28 ± 0.87	0.0063
Ratio insulin/glucose	0.10 ± 0.18	0.15 ± 0.02	0.0600
HOMA IR index ^1^	0.40 ± 0.09	0.87 ± 0.07	0.0001
TG (mg/dL)	95.00 ± 4.98	112.44 ± 6.07	0.0347
Total cholesterol (mg/dL)	112.39 ± 4.24	105.91 ± 5.38	0.3500
Ratio TG/cholesterol	0.85 ± 0.05	0.98 ± 0.06	0.0940
AST (U/L)	33.27 ± 2.55	33.28 ± 2.84	0.9977
IGF1 (ng/mL)	123.88 ± 7.45	140.82 ± 8.92	0.1564

^1^: HOMA index: [insulin (mU/L) × (glucose (mg/dL)/18)]/22.5; TG: Triglycerides; ALT: serum alanine transaminase; AST: serum aspartate transaminase, IGF1: insulin growth factor 1.

## Data Availability

The data presented in this study are available on request from the corresponding author.

## Supplementary Materials

The following are available online at https://www.mdpi.com/2072-6643/13/2/310/s1. Figure S1. Sirius red staining in livers of female offspring from dams (a) fed ad libitum (Control group); (b) fed restricted (105 g/day) during the first two-thirds of gestation and re-fed ad libitum (R021 group).
