# Supplementary material for: Gestation Food Restriction and Refeeding Compensate Maternal Energy Status and Alleviate Metabolic Consequences in Juvenile Offspring in a Rabbit Model
Source: Nutrients. 2021 Jan 22;13(2):310. doi: 10.3390/nu13020310 (PMC7912334; doi:10.3390/nu13020310)
Supplement: Supplementary file 1 [file nutrients-13-00310-s001.pdf]

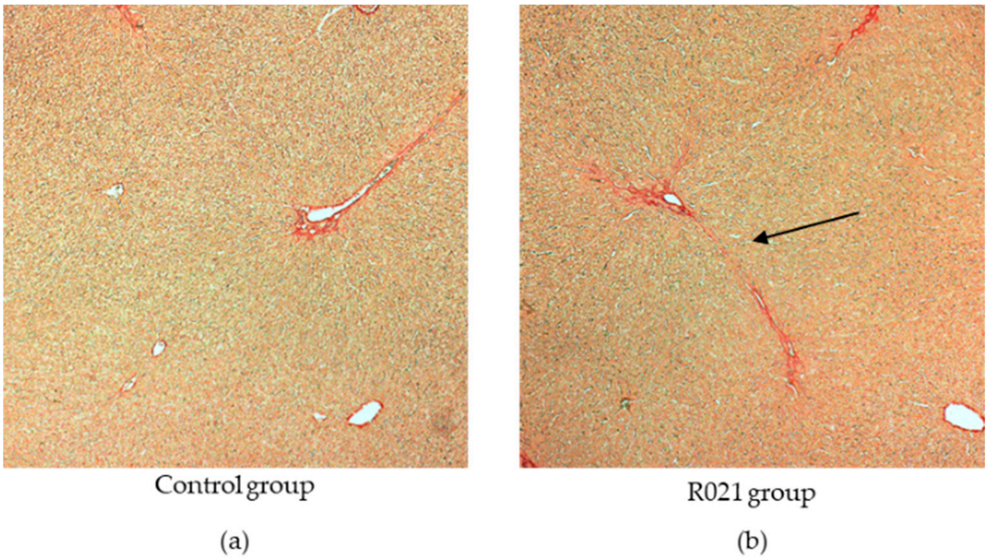

Figure S1. Sirius red staining in livers of female offspring from dams (**a**) fed ad libitum (control group); **(b)** fed restricted (105 g/day) during the first two-thirds of gestation and re-fed ad libitum (R021 group). Arrow indicates septum formation. Magnification 10×.
